# Impact of stent length and diameter on short term clinical outcomes of drug eluting stents in patients with stable coronary artery disease

**DOI:** 10.12669/pjms.334.13068

**Published:** 2017

**Authors:** Yasir Adnan, Lubna Noor, Muhammad Habeel Dar, Umair Ali, Muhammad Hafizullah

**Affiliations:** 1 Dr. Yasir Adnan, FCPS Cardiology, Department of Cardiology, Police and Services Hospital, Peshawar, Pakistan; 2 Dr. Lubna Noor, FCPS Cardiology, Department of Cardiology, Lady Reading Hospital, Peshawar, Pakistan; 3 Dr. Muhammad Habeel Dar, FCPS Cardiology, Department of Cardiology, Lady Reading Hospital, Peshawar, Pakistan; 4 Dr. Umair Ali, FCPS Cardiology, Department of Cardiology, Lady Reading Hospital, Peshawar, Pakistan; 5 Dr. Muhammad Hafizullah, FRCP, Department of Cardiology, Lady Reading Hospital, Peshawar, Pakistan

**Keywords:** Coronary Artery Disease, Drug Eluting stent, Exercise Tolerance Test

## Abstract

**Background & Objective:**

The risk of restenosis and other adverse cardiovascular events with bare-metal stents have increased with smaller stent diameters and longer stent lengths. However, the exact impact of stent size on the short-term outcomes of drug-eluting stent (DES) implantations has not been much classified in Pakistani population. This study was designed to evaluate the impact of size (length and diameter) of Drug Eluting Stents on Clinical outcomes in patient with stable coronary artery disease at three months of implantation in Pakistani Population.

**Methods:**

This is a prospective study which was carried out in the Department of Cardiology, Lady Reading Hospital Peshawar from April 2011 and July 2012. All consecutive patients with stable coronary disease undergoing Percutaneous Intervention (PCI) with DES implantation at Cardiology Unit Lady Reading Hospital, were included prospectively. Clinical outcomes (Myocardial infarction [MI], unstable angina[UA], and positive ETT) at three months stratified by 3 tertiles of stent length and diameter each, were measured in patients who underwent PCI with DES for coronary artery lesions. All patients were followed and reassessed after three months from the index procedure. Exercise Tolerance Test(ETT) was performed on every patient and recorded on proforma. Data analysis was done using SPSS software version 16.

**Results:**

A total of 376 patients were included prospectively in this study. The mean age was 57±9.313 years. Male patients were 271(72.1%). Mean length of drug eluting stent was 27.313±7.235 mm while mean diameter of stent was 2.90±0.2483mm. There were slightly higher rates of MI, U.A and positive ETT in the longest stent length tertile(>28mm) compared with the shortest stent length tertile (<22mm) at three months, but they were statistically not significant. We also observed that for DES, there was no clear relationship between stent diameter and outcome for any of the clinical outcome variables.

**Conclusion:**

In our single-center prospective study, stent length and diameter defined in tertiles, had no impact on the short-term clinical outcomes of DES in patients with stable coronary artery disease.

## INTRODUCTION

The management of long coronary lesions by percutaneous coronary intervention (PCI) has become increasingly important because of the rising incidence of long or complex lesions in aging populations.^1^ Long lesions account for approximately 20% of PCI and present great challenges for drug-eluting stenting.^2^-^4^ Management of long lesions with single long stent is a preferred strategy of PCI. Stent length has been considered an important predictor of adverse events after PCI. Long lesions have been associated with adverse outcomes in percutaneous coronary interventions treated with bare metal stents (BMS).^5^ However, the exact impact of lesion length on the short- and long-term clinical outcomes of drug-eluting stent (DES) implantations is not clear as yet.

Among the most common off-label uses of drug-eluting stents (DESs) is their placement to treat long lesions or diffuse coronary artery disease. In pivotal DES approval trials, longer stent length was associated with an increased rate of adverse clinical events.^6^,^7^ However, because these trials employed strict inclusion/exclusion criteria, limits to stented segment length, routine angiographic follow-up and limited to western populations, the specific impact of DES length in routine clinical practice is not well established in our local patients.

There are only limited data examining the effect of stent length and diameter with DES in routine clinical practice including use in patients and for lesions that would not have been included in randomized clinicaltrials.^8^,^9^ We, therefore, undertook this study to evaluate the effect of DES length and diameter on clinical outcome in patients with Stable Coronary Artery Disease (SCAD) in Pakistani population.

## METHODS

This prospective study was carried out in the Department of Cardiology, Lady Reading Hospital Peshawar from April 2011 and July 2012. The total study duration was 15 months. A total of 376 patients underwent PCI with DES. All patients of stable angina pectoris of any age and sex who were treated with Drug Eluting Stents irrespective of the lesion length were included in the study. Patients with previous history of revascularization, whether primary or elective PCI or Coronary Artery Bypass Graft (CABG) and patients having any contraindication to ETT were excluded. Patients with Left main stem disease or triple vessel disease on coronary angiography were also excluded.

Clinical outcomes (Myocardial infarction [MI], unstable angina [UA], andpositive ETT) at three months stratified by three stent length tertiles and diameter tertiles were measured in patients who received DES for coronary artery lesions. Use of Drug Eluting Stents via radial or femoral routes in all patients from both genders of any age, stent size, stent diameter and stented coronary vessels was documented on a specified proforma.

### Operational definitions of three months clinical outcomes unstable angina

This was defined as history of chest pain which occurs at rest, without provocation, crescendo pattern in frequency, duration or intensity accompanied by ST segment depression more than 1mm in at least two consecutive leads or symmetrical T wave inversion ≥ 2mm in the same leads and normal serum troponin-I(<0.14 ng/ml) via 3rd generation enzyme linked immunosorbent assay (ELISA) using Architect (I 2000 SR).

### Positive exercise tolerance test

It was defined as exercise tolerance test positive for ischemia (down sloping or horizontal 1mm ST segment depression) or angina using Bruce protocol.

### Myocardial Infarction

This was defined as the presence of two of the following.

- Prolonged chest pain more than 20 minutes not relieved by rest.
- Cardiac enzyme elevation with raised Troponin I (> 0.14ng/ml) via 3rd generation enzyme linked immunosorbent assay (ELISA) using Architect (I 2000 SR).
- ST-segment elevation of more than 1mm in consecutive leads or ST segment depression of more than 1mm in consecutive leads or symmetrical T wave inversion ≥ 2mm in the same leads or new Q waves on serial electrocardiograms indicative of myocardial damage.

All patients who underwent PCI (DES stent) for stable angina pectoris were called back for follow-up and reassessed after three months from the index procedure. History was taken regarding unstable angina, myocardial infarction and hospitalization for any of these events over the last three months. ETT was performed on every patient and was recorded on proforma. Study exclusion criteria were followed to control confounders and bias in the study results. Data analysis was done using SPSS version 16.

## RESULTS

A total of 376 patients were included in this study. Patients were followed for three months. Mean age was 57±9.313. Male patients were 271 (72.1%) while female were 105 (27.9%). Majority of the patients got stented to left anterior descending (LAD) and left circumflex (LCX) artery. Mean length of drug eluting stent was 27.313±7.235 while the mean diameter was 2.90±0.2483 (Table-I). Complete three months follow-up was available in all of the patients of the study population. Most of the patients in each tertile were hypertensive (i.e. about 70%), whereas majority of patients in each length tertile got stented to LAD and LCX arteries.

**Table-I:** Overall Patients demographic data and angiographic characteristics. Overall (n = 376).

Age	57±9.313
Male	72.1% (271)
***Target vessel***	
LAD	46.8% (176)
LCX	27.9% (105)
RCA	6.4% (24)
LAD and LCX	9.8% (37)
LCX and RCA	6.1% (23)
LAD and RCA	2.9% (11)
Mean Length of stent	27.314±7.234
Mean Diameter of stent	2.903±0.248
Hypertensive	260 (69.1%)
Diabetic	144 (38.3%)
Smokers	70 (18.6%)
Dyslipidemia	165 (43.9%)

When patients were stratified among male and female, there was a trend towards increase events rate in female patients but it was statistically insignificant. (Table-II)

**Table-II:** Clinical outcomes among male and female patients. Overall (n = 376)

*Gender*	*Male (n=271)*	*Female (n=105)*	*P-value (Two sided)*
Myocardial Infarction	2.6% (7)	3.8% (4)	0.509
Unstable Angina	6.3% (17)	8.6% (9)	0.497
Positive ETT	9.6% (26)	10.5% (11)	0.847

Patients were stratified into 3 tertiles according to stent length. We measured the rate of selected outcomes (Myocardial infarction [MI], unstable angina [UA], and positive ETT) at three months (Table-III). As the tertile of stent length increased, there was a trend toward higher rates of clinical outcomes. There were slightly higher rates of MI, U.A and positive ETT in the longest stented length tertile (>28mm) compared with the shortest stent length tertile (<22mm) at three months, but they were statistically not significant.

**Table-III:** Effect of stent length on clinical outcomes. Overall (n=376).

*Stent length (in mm)*	*<22mm (n=72)*	*22-28mm (n=146)*	*>28mm (n=158)*	*p-value*
MI	1.4% (1)	1.4% (2)	5.1% (8)	0.112
U.A	4.2% (3)	5.5% (8)	9.5% (15)	0.229
Positive ETT	8.3% (6)	8.9% (13)	11.4% (18)	0.685

We also stratified patients undergoing DES implantation into three tertiles according to diameter of stent. The rate of clinical outcomes (Myocardial infarction [MI], unstable angina [UA], and positive ETT) at three months were measured. The event rate of selected outcomes for stented diameter tertiles at three months is shown for DES in Table-IV. For DES there were no clear relationships between stented diameter and outcome for any of the clinical outcome variables (Table-III).

**Table-IV:** Effect of stent diameter on clinical outcomes. Overall (n=376).

*Stent diameter (in mm)*	*<3mm (n=172)*	*3-3.4mm (n=172)*	*>3.4mm (n=32)*	*p-value*
MI	2.3% (4)	3.5% (6)	3.1% (1)	0.903
U.A	7.6% (13)	6.4% (11)	6.2% (2)	0.790
Positive ETT	12.2% (21)	6.4% (11)	15.6% (5)	0.100

## DISCUSSION

We have presented three months data of clinical outcomes of the Drug Eluting Stents stratified by stent length and diameter in patients with stable coronary artery disease. These results, obtained from 376 patients, provide compelling evidence for the safe and effective use of the DES in lesions of any length or diameter. All reported events were adjudicated. The three months clinical outcomes included MI, U.A and positive ETT.

In the BMS era, increased stent length was identified as a powerful and consistent predictor of restenosis.^10^-^12^ Similarly, stent length and lesion length have been reported as independent predictors of IRS in various DES such as sirolimus-eluting stents.^13^-^15^ However, in the present study we did not see the same result. According to our data, stent length has no significant effect on event rate for DES though there was a trend towards higher rate of events with increasing stent length. This might be due to short term follow up in our study as most of the trials did follow their patients for more than one year, which explains the higher incidence of MACE and restenosis rate. In a recent single-center prospective registry, lesion length defined in tertiles has no impact on the short-term (ISR) or long-term (MACE) outcomes of patients implanted with DES. DES considerably lowers the effects of lesion length on ISR rates and MACE-free survival. In contrast, longer lesion correlates with higher ISR and MACE rates in BMS group.^5^ The latest Bioresorbable stents (BRS) are an emerging technology for the treatment of coronary artery stenosis. No significant differences were detected regarding overall clinical outcome and rates of stent thrombosis after one year, in relation to the size of the implanted Bioresorbable stents (BRS).^16^

We also stratified the patients into three tertiles according to stent diameter. We observed that for DES there were no clear relationships between stented diameter and outcome for any of the clinical outcome variables. In general, the treatment of lesions in small vessels is more challenging.^17^,^18^ Firstly, due to smaller reference vessel size, the likelihood of restenosis is higher, since the same proportion of neointimal tissue growth in comparison to vessels with a larger diameter is more likely to lead to a clinically relevant restenosis. Secondly, patients with small-vessel disease more often suffer from diabetes mellitus and tend to have longer and more complex lesion morphology.^17^ However In later studies, the benefit of DES over bare metal stents was clearly demonstrated and thus DES were considered to be the optimal treatment strategy for small vessels.^19^

### Study limitations

An important limitation of this study is the potential of underreporting of adverse events. Several measures, including ongoing random monitoring of patients enrolled and adjudication of events by review electrocardiogram and laboratory values for MI and U.A, were employed to minimize this occurrence in this study, but the inherent possibility exists for inaccuracy of reported outcomes. Another limitation of this study was that no regular angiographic follow up was done.

## CONCLUSION

Our observations of lower rates of MI, UA and positive ETT at three months in DES-treated patients of stable coronary artery disease across a wide range of stented lengths and diameters is reassuring that routine use of DES is safe and effective for both long (>28 mm) and large (>3.4 mm) lesions. Further research is necessary to ascertain long-term safety of drug-eluting stents across a wide range of stent length and diameter and to identify additional factors to promote long term efficacy and safety of DES.

### Authors’ Contribution

**YA** conceived, designed and did statistical analysis.

**LN** did editing of manuscript.

**LN, MHD and UA** did data collection and manuscript writing.

**MH** did review and final approval of manuscript.
